# Dexketoprofen Pharmacokinetics is not Significantly Altered by Genetic Polymorphism

**DOI:** 10.3389/fphar.2021.660639

**Published:** 2021-04-29

**Authors:** Gina Mejía-Abril, Pablo Zubiaur, Marcos Navares-Gómez, Gonzalo Villapalos-García, Manuel Román, Dolores Ochoa, Francisco Abad-Santos

**Affiliations:** ^1^ Clinical Pharmacology Department, Hospital Universitario de La Princesa, Instituto Teófilo Hernando, Universidad Autónoma de Madrid (UAM), Instituto de Investigación Sanitaria La Princesa (IP), Madrid, Spain; ^2^ UICEC Hospital Universitario de La Princesa, Plataforma SCReN (Spanish Clinical Research Network), Instituto de Investigación Sanitaria La Princesa (IP), Madrid, Spain; ^3^ Centro de Investigación Biomédica en Red de Enfermedades Hepáticas y Digestivas (CIBERehd), Instituto de Salud Carlos III, Madrid, Spain

**Keywords:** dexketoprofen, pharmacogenetics, cytochrome P450, precision medicine, ABC transport protein

## Abstract

Dexketoprofen is the (S)-(+)-enantiomer of racemic ketoprofen, a nonsteroidal anti-inflammatory drug used for the management of different types of pain. To the best of our knowledge, no article was published to date on dexketoprofen pharmacogenetics. Thence, in this work, we aimed to explore the influence of sex, race and several single nucleotide polymorphisms (SNPs) in genes encoding metabolizing enzymes (e.g. *CYP* or *UGT*) or transporters (e.g., *ABC or SLC*) in the pharmacokinetics and safety of dexketoprofen to explore whether dosing adjustments based on genetic polymorphism would be beneficial for its prescription. For this regard, 85 healthy volunteers enrolled in three bioequivalence clinical trials were genotyped for 46 SNPs in 14 genes. Women showed lower AUC adjusted by dose/weight (AUC/DW) and higher Vd/F and Cl/F than men (*p* < 0.05 in univariate and multivariate analysis). CYP1A2*1B allele, *CYP2B6* IM/PM and *CYP2D6* IM/PM phenotypes were related to drug accumulation (AUC/DW or Cmax/DW) compared to the CYP1A2*1 allele, *CYP2B6* NM/RM and *CYP2D6* NM/UM phenotypes (*p* < 0.05 in the univariate analysis). *ABCB1* C1236TT, C3435TT and G2677A/TA/T alleles were related to lower Cmax/DW compared to C, C, and G alleles (*p* < 0.05 in univariate and multivariate analysis). *ABCB1* C1236TT allele was also related to lower AUC/DW (*p* < 0.05 in multivariate analysis). The remaining studied transporter genes (*ABCC2*, *SLC22A1*, and *SLCO1B1*) and metabolizing enzyme genes (*CYP3A5*, *CYP2C19*, *CYP2C9*, *CYP2C8*, *CYP3A4*, *CYP2A6*, and *UGT1A1*) were unrelated to dexketoprofen pharmacokinetic variability. We conclude that dexketoprofen pharmacokinetics can be influenced by several polymorphisms, although there is not a clear pharmacogenetic predictor that would justify individualization of therapy based on its genotyping. Further studies should be conducted to confirm the role of SNPs in *CYP2B6*, *CYP2D6*, *CYP1A2* and *ABCB1* on the pharmacokinetic variability of dexketoprofen. Current evidence on dexketoprofen pharmacogenetics does not justify its inclusion in pharmacogenetic guidelines.

## Introduction

Racemic ketoprofen is a chiral 2-arylpropionic acid derivative nonsteroidal anti-inflammatory drug (NSAID) with anti-inflammatory and antipyretic properties. The (S)-(+)-enantiomer (dexketoprofen) is active, while (R)-(−)-ketoprofen is completely inactive (Barbanoj et al., 2001). Dexketoprofen is used for the management of mild to moderate intensity pain, e.g., musculoskeletal pain, dysmenorrhea or odontalgia.

Maximum daily dose is 75 mg, being 25 mg/8 h, 12.5 mg/4 h or 12.5 mg/6 h the recommended doses. In elderly patients, or those with hepatic or renal impairment, the maximum daily dose should be reduced and patients should be closely monitored. Administered as a water-soluble salt (dexketoprofen trometamol), dexketoprofen reaches the maximum concentration (Cmax) 30 min (15–60 min) after drug intake; absorption is delayed by the presence of food. It binds extensively to plasmatic proteins (99%) with a mean volume of distribution of 0.25 L/kg. It is extensively glucuronized to conjugates and eliminated in urine in this form (Barbanoj et al., 2001; Agencia Española del Medicamento y Productos Sanitarios (AEMPS), 2021).

To the best of our knowledge, no article was published to date on dexketoprofen pharmacogenetics. However, understanding predictors of the effectiveness and safety of dexketoprofen would help to personalize treatment, as reported for other NSAIDs like ibuprofen or meloxicam (Theken et al., 2020). The most relevant observed adverse drug reactions (ADRs) to dexketoprofen are gastrointestinal. These include nausea, vomiting (frequent), abdominal pain, flatulence, constipation, dyspepsia, diarrhea, mouth dryness, hematemesis (infrequent) and other less frequent ADRs like peptic ulcers with perforation or gastrointestinal bleeding (Barbanoj et al., 2001). These can lead to treatment discontinuation and improper management of patient pain.

On March 2020, the Clinical Pharmacogenetics Implementation Consortium published their guideline on Nonsteroidal Anti-inflammatory Drugs and CYP2C9 (Theken et al., 2020). This included dosing recommendations for ibuprofen and other NSAIDs based on CYP2C9 phenotype. However, no recommendation or information was included regarding dexketoprofen, probably due to the lack of pharmacogenetic information available nowadays. Hence, in this work, we aimed to explore the influence of several single nucleotide polymorphisms (SNPs) in genes encoding metabolizing enzymes (e.g., *CYP* or *UGT*) or transporters (e.g., *ABC or SLC*) in the pharmacokinetics and safety of dexketoprofen to explore whether dosing adjustments based on genetic polymorphism would be beneficial for its prescription.

## Materials and Methods

### Study Population

Healthy volunteers enrolled in three dexketoprofen bioequivalence clinical trials (EUDRA-CT: 2011-002728-42, 2011-003800-19 and 2019-002223-15. Ethics Committee approval numbers 1815, 1861 and 3818 respectively) conducted in the Clinical Trial Unit of Hospital Universitario de La Princesa (UECHUP), Madrid, (Spain) participated in the pharmacogenetic study. Hospital’s Research Ethics Committee and the Spanish Drugs Agency (AEMPS) revised and approved the study protocols; research was conducted complying with European and Spanish legislation and under Good Clinical Practice Guidelines; the Declaration of Helsinki (revised) was fully endorsed. All healthy volunteers (*n* = 96) signed their informed consent for their participation in the clinical trials, among which, 85 signed a specific informed consent for the pharmacogenetic study.

Inclusion criteria for the participation in the clinical trial were as follows: males or females aged 18 to 55, free from psychic conditions, with normal physical examination and medical records, without abnormalities in hematology, biochemistry, serology and urine tests. Exclusion criteria comprised any organic or psychic condition, having received prescribed pharmacological treatment in the last 15 days or any type of medication in the 48 h prior to study medication intake, body mass index (BMI) out of the 18.5–30 kg/m^2^, history of drug sensitivity, suspected consumption of controlled substances, smoking, alcohol poisoning in the last week or daily alcohol consumption, having donated blood in the last month and pregnant or breastfeeding women.

### Study Design and Procedures

The three clinical trials were phase I, oral single-dose, open-label, crossover and randomized, organized in 2 sequences and 2 periods evaluating bioequivalence between dexketoprofen 25 mg test formulations and Enantyum 25 mg (Menarini, Spain). Volunteers were hospitalized either the night before or 1 h before drug intake in the morning and remained hospitalized 12 h after dosing. Between drug intake (0 h), and 12 h after dosing, 17 or 18 blood samples were extracted for pharmacokinetic profiling, which was blinded. Formulations were administered by oral route with 240 ml of water under fasting conditions. Blood was collected in EDTA-K2 tubes; plasma was separated immediately after each blood extraction by centrifugation and samples were frozen until their shipment to the analytical laboratory. The method involved a liquid-liquid extraction procedure with tert-butyl methyl ether; dexketoprofen and internal standard were measured by reversed phase high performance liquid chromatography coupled to a tandem mass spectrometer (LC/MS/MS). Method complied with the European Medicines Agency (EMA) requirements for bioequivalence demonstration.

### Pharmacokinetic Analysis

The CERTARA Phoenix WinNonlin software, most recent version at the moment of clinical trial performance (Certara United States, Princeton, NJ, United States) was used for the non-compartmental analysis of pharmacokinetic parameters. The area under the curve (AUC) from pre-dose to the last time-point (12 h) was calculated following the trapezoidal rule (AUC_t_). By means of linear regression of the log-linear part of the concentration-time curve, the terminal rate constant (k_e_) was calculated. The AUC from t = 12 to infinite was calculated as C_t_/k_e_ (AUC_t-∞_). AUC_∞_ was calculated as: AUC_t_ + AUC_t-∞_. By dividing the dose (D) by AUC_∞_ and weight, drug clearance adjusted for bioavailability (Cl/F) was calculated. By dividing Cl/F by k_e_, the volume of distribution adjusted for bioavailability (Vd/F) was calculated. Half-life (t_1/2_) was estimated as–ln 2/k_e_. The maximum concentration (C_max_) and the time to reach the C_max_ (t_max_) were directly obtained from concentration-time curves.

### Safety

Abnormalities in analytical values, blood parameters, physical examinations or any other clinically relevant event were noted for safety assessment. A 12-lead electrocardiogram (ECG) was carried out at predose and 1 or 2 h after drug intake. Simultaneously, vital signs were monitored (systolic and diastolic blood pressure, heart rate and tympanic or armpit temperature). Volunteers were monitored for the occurrence of adverse events (AEs) by an open question, and, in addition, those reported spontaneously by themselves were noted. The Spanish Pharmacovigilance System (Aguirre and García, 2016) algorithm or Karch and Lasagna criteria (Karch and Lasagna, 1977) were used for causality determination. Only those AEs with a definite, probable or possible causality were defined as adverse drug reactions (ADRs) and therefore considered for analysis.

### Genotyping, Haplotyping and Phenotyping

A MagNa Pure automatic DNA extractor (Roche Applied Science, United Status) was used for the extraction of DNA form peripheral blood collected in an EDTA-K2 tube. 46 SNPs in 14 genes were analyzed: CYP1A2*1C (rs2069514), *1F (rs762551), *1B (rs2470890), CYP2A6*9 (rs28399433), CYP2B6*9 (rs3745274), *5 (rs3211371), rs4803419, rs2279345, rs2279343, CYP2C8*2 (rs11572103), *3 (rs10509681), *4 (rs1058930), CYP2C9*2 (rs1799853), *3 (rs1057910), CYP2C19*2 (rs4244285), *3 (rs4986893), *4 (rs28399504), *17 (rs12248560), CYP2D6*3 (rs35742686), *4 (rs3892097), *6 (rs5030655), *7 (rs5030867), *8 (rs5030865), *9 (rs5030656), *10 (rs1065852), *14 (rs5030865), *17 (rs28371706), *41 (rs28371725), *CYP3A4* *22 (rs35599367), rs55785340, rs4646438, CYP3A5*3 (rs776746), *6 (rs10264272), *ABCB1* C3435T (rs1045642), G2677 T/A (rs2032582), C1236T (rs1128503), *ABCC2* c.1247G>A (rs2273697), rs717620, SLCO1B1*1B (rs2306283), *5 (rs4149056), c.-910G > A (rs4149015), rs11045879, SLC22A1*2 (rs72552763), *3 (rs12208357), *5 (rs34059508), and UGT1A1*80 (rs887829). A *CYP2D6* copy number variation assay (CNV) was performed following the methodology previously reported (Zubiaur et al., 2020). For the genotyping, a QuantStudio 12 k flex was used with two thermal blocks: the OpenArray for the genotyping of variants and the 96-well fast thermal-block for CNV determination (Zubiaur et al., 2020).

*CYP2D6* (*3, *4, *5, *6, *7, *8, *9, *10, *14, *17, *41 and the gene copy number)*, CYP2C19* (*2, *3, *4, *17), *SLCO1B1* (*1B, *5), *CYP2B6* (*5 and *9) and *CYP2C9* (*2, *3) variants were used to infer the enzymatic phenotype based on CPIC guidelines (Scott et al., 2013; Caudle et al., 2014; Ramsey et al., 2014; Caudle et al., 2019; Desta et al., 2019). For phenotype inference, those variants lacking a result were considered “not mutated.” Missing data due to genotyping errors (e.g., absence of amplification) were not analyzed. *CYP1A2* (*1C, *1F and *1B) variants were used to infer the activity score and phenotype as described in previous publications (Saiz-Rodríguez et al., 2019; Zubiaur et al., 2019). *SLC22A1* and *ABCB1* variants were merged into haplotypes: the absence of any variant was assigned the wild-type haplotype, the presence of one variant was assigned the heterozygous haplotype and the presence of two or more variants was assigned the mutant haplotype.

### Statistical Analysis

The SPSS software was used for statistical analysis (version 19.0, SPSS Inc., Chicago, United States). AUC_∞_ and C_max_ were divided by the dose/weight ratio (AUC/DW, C_max_/DW) to correct the differences in weight between sexes or races which can produce pharmacokinetic variability. For distribution normalization, pharmacokinetic parameters were logarithmically transformed. Firstly, a univariate analysis was performed. The means of pharmacokinetic parameters or the incidence of adverse drug reactions (ADRs) were compared based on categorical variables (e.g., sex, race, haplotypes, phenotypes). For mean comparison, a T test (variables with two categories) or an ANOVA test followed by a Bonferroni post-hoc (variables with three or more categories) were used. For the comparison of the incidence of ADRs according to categorical variables, a Chi^2^ test was used. Afterward, each pharmacokinetic parameter or ADR were individually analyzed with a multivariate analysis. By linear or logistic regression, pharmacokinetic parameters or ADRs were explored, respectively. As independent variables, any variable with *p* < 0.05 in the univariate analysis was explored (sex, race, *ABCB1* C1236T, C3435T, G2677A/T, CYP1A2*1B, *CYP2B6*, and *CYP2D6* phenotypes); in addition, pharmacokinetic parameters were introduced as independent variables in the logistic regression for the evaluation of ADR occurrence.

## Results

### Demographic Characteristics

Study population was composed by 42 women (49.41%) and 43 men (50.59%). Men’s height, weight and body mass index (BMI) were significantly superior than that of women (*p* < 0.0001, *p* < 0.0001, and *p* = 0.030, respectively) (Table 1). Caucasian was the most prevalent race (82%) compared to Latin-Americans (15%), one Black and one Asian (both males). Demographics also differed significantly according to races (Table 1). Caucasians were younger than Latin-Americans (*p* < 0.003).

**TABLE 1:** Demographic characteristics of the study population.

Sex	*n*	Age (years)	SD	Weight (kg)	SD	Height (m)	SD	BMI (kg/m^2^)	SD
Men	43	26.65	7.09	74.70	9.63	1.77	0.07	23.71	2.45
Women	42	26.67	8.42	59.52*	8.03	1.63*	0.06	22.46*	2.77
Race									
Caucasian	70	25.66	6.59	67.55	11.88	1.71	0.10	22.98	2.62
Latin-American	13	**33.08** ^#^	10.52	64.91	10.51	1.65	0.08	23.80	3.00
Black	1	19.00	—	57.90	—	1.68	—	20.51	—
Asian	1	21.00	—	81.70	—	1.82	—	24.66	—
Total	85	26.66	7.73	67.20	11.67	1.70	0.10	23.10	2.67

*: *p* < 0.05 after T-test comparing men with women; ^#^: *p* < 0.05 after T-test comparing Caucasian with Latin-American (Black and Asian were not included in this analysis because of the low number of subjects).

The bold values simply serve to highlight significant results.

### Pharmacokinetics

Dexketoprofen mean AUC_∞_ was 4,031.88 ± 819.35 ng*h/mL (4,266.90 ± 832.59 ng*h/mL for females and 3,802.33 ± 746.18 ng*h/mL for males, *p* = 0.008) and mean C_max_ was 2,745.20 ± 690.81 ng/ml (3,013.30 ± 726.25 ng/ml for females and 2,483.34 ± 545.43 ng/ml for males, *p* < 0.001). After DW correction, women showed lower AUC/DW compared to men (ANOVA *p* = 0.007, unstandardized beta coefficient = −0.124, *p* = 0.011, model *R* ^2^ = 0.151) and the differences in Cmax/DW disappeared (Table 2). The Black and Asian subjects showed higher AUC/DW, Cmax/DW and half-life and lower Cl/F and Vd/F, but statistical analysis was not possible. Latin-Americans showed lower AUC/DW and t_1/2_ than Caucasians (Table 2); however, these associations disappeared after the multivariate analysis. Lastly, women exhibited higher Vd/F and Cl/F than men (unstandardized beta coefficients = 0.114 and 0.125; *p* = 0.012 and 0.010; model *R* ^2^ = 0.097 and 0.155, respectively).

**TABLE 2:** Dexketoprofen pharmacokinetic parameters based on sex and race.

		N	AUC/DW (kg*h*ng/mL*mg)	SD	Cmax/DW (kg*ng/mL*mg)	SD	Tmax (h)	SD	T1/2 (h)	SD	Vd/F (ml/kg)	SD	Cl/F (mL/h*kg)	SD
Sex	Male	43	11,253.32	2,233.52	7,328.16	1,467.24	0.62	0.31	2.27	0.35	297.69	47.36	92.36	17.10
	Female	42	10,032.45*	1822.22	7,122.11	1730.30	0.76	0.53	2.25	0.37	331.29*	59.57	103.20*	18.42
Race	Caucasian	70	10,776.95	2097.40	7,210.42	1,567.78	0.71	0.46	2.30	0.37	315.75	58.72	96.52	18.67
	Latin—american	13	**9,481.61** ^#^	1,209.83	7,053.52	1732.39	0.62	0.33	**2.02** ^#^	0.19	310.28	32.70	**107.26** ^#^	12.82
	Black	1	10,849.29	—	7,810.99	—	0.50	—	2.70	—	364.42	—	94.13	—
	Asian	1	16,758.60	—	10,003.27	—	0.42	—	2.46	—	214.20	—	60.59	—

SD: standard deviation; *: after T-test comparing men with women; ^#^: *p* < 0.05 after T-test comparing Caucasian with Latin-American (Black and Asian were not included in this analysis because of the low number of subjects).; Underlined: *p* < 0.05 after multivariate analysis (linear regression, which included the following variables: sex, race, CYP2B6 phenotype, CYP2D6 phenotype, CYP1A2*1B, ABCB1 C3435T (rs1045642), G2677 T/A (rs2032582), and C1236T (rs1128503)).

The bold values simply serve to highlight significant results.

Some genetic polymorphisms were associated with dexketoprofen pharmacokinetic variability (Table 3). The CYP1A2*1B/*1B genotype was related to higher Cmax/DW (ANOVA *p* = 0.035) and to lower Tmax (ANOVA *p* = 0.022, unstandardized beta coefficient = −0.177, *p* = 0.015, *R* ^2^ = 0.163) compared to the *1/*1 genotype (Table 3) and to higher Tmax compared to the *1/*1B genotype (*p* = 0.050). *CYP2B6* intermediate (IM) and poor (PM) metabolizers were related to higher Cmax/DW compared to normal (NM) and rapid (RM) metabolizers (ANOVA *p* = 0.023 and 0.037, respectively). These associations were lost after multivariate analysis. Moreover, *CYP2B6* intermediate and poor metabolizers were related to lower Tmax compared to RMs (ANOVA *p* = 0.024, unstandardized beta coefficient = −0.130, *p* = 0.043, *R* ^2^ = 0.163). *CYP2D6* IMs/PMs were related to higher AUC_∞_/DW compared to NMs (*p* = 0.017). This association was not confirmed by multivariate analysis.

**TABLE 3:** Significant associations between dexketoprofen pharmacokinetic parameters and genotypes or phenotypes of CYP enzymes and the ABCB1 transporter.

		N	AUC/DW (kg*h*ng/mL*mg)	SD	Cmax/DW (kg*ng/mL*mg)	SD	Tmax (h)	SD	T1/2 (h)	SD	Vd/F (ml/kg)	SD	Cl/F (mL/h*kg)	SD
CYP1A2*1B	*1/*1	25	10,887.13	1968.93	6,606.77	1,565.26	0.88	0.65	2.28	0.38	309.56	58.22	95.35	18.34
	*1/*1B	41	10,757.90	2,408.75	7,426.17	1,535.04	0.63	0.31	2.26	0.34	311.79	53.51	97.54	20.40
	*1B/*1B	18	10,175.26	1,590.83	7,774.75* ^**4**^	1,489.90	0.55* ^**5**^	0.15	2.28	0.39	327.40	61.18	100.50	14.20
*CYP2B6* phenotype	RM	22	10,482.63	1990.54	6,701.00	1,381.54	0.83	0.57	2.15	0.30	304.47	59.34	99.02	18.63
	NM	21	10,561.54	2,309.80	6,739.11	1853.38	0.78	0.56	2.35	0.44	327.84	56.91	98.67	20.10
	IM/PM	40	10,881.81	2,132.90	7,762.43* ^**1**^	1,437.57	0.57* ^**2**^	0.21	2.29	0.33	311.83	54.28	95.68	17.93
*CYP2D6* phenotype	UM	15	10,545.05	2084.50	7,046.11	1,603.15	0.86	0.81	2.25	0.28	317.61	59.98	98.32	17.53
	NM	42	10,226.92	1974.41	7,111.57	1,637.38	0.67	0.35	2.17	0.36	312.48	43.80	101.61	18.99
	IM/PM	22	11,727.63* ^**3**^	2,199.68	7,411.46	1,567.68	0.63	0.23	2.39	0.39	301.46	64.44	88.11*^3^	14.52
*ABCB1* C1236T rs1128503	CC	22	11,271.67	1943.23	7,777.70	1,522.11	0.61	0.20	2.34	0.28	307.11	58.90	**91.29**	14.94
	CT	39	10,751.84	2,560.98	7,354.49	1,595.90	0.68	0.47	2.26	0.42	313.99	61.98	98.54	22.61
	TT	12	**9,845.04**	1,112.75	6,102.44* ^**6**^	1,581.93	0.83	0.61	2.33	0.36	343.77	46.48	103.09	12.28
*ABCB1* C3435T rs1045642	CC	22	10,795.42	2,150.38	7,755.61	1,547.70	0.60	0.19	2.27	0.33	310.98	58.30	96.04	17.27
	CT	40	10,760.74	2,378.71	7,270.56	1,548.30	0.68	0.51	2.24	0.37	309.22	55.41	97.79	21.19
	TT	22	10,252.73	1,601.75	6,513.63* ^**7**^	1,507.43	0.80	0.46	2.30	0.38	328.74	55.61	99.82	14.79
*ABCB1* G2677A/T rs2032582	GG	18	10,942.11	1738.26	7,675.95	1,493.76	0.61	0.19	2.29	0.35	307.75	56.19	93.86	15.36
	GT/GA	48	10,685.84	2,516.64	7,397.63	1,553.87	0.67	0.47	2.23	0.36	310.93	55.91	98.81	21.58
	TT/AA	18	10,200.57	1,132.67	6,194.02* ^**8**^	1,399.29	0.84	0.51	2.33	0.39	332.15	56.50	99.34	11.51

SD: standard deviation; *1: *p* < 0.05 after ANOVA and Bonferroni post-hoc (IM/PM vs. RM and NM); *2: *p* < 0.05 after ANOVA and Bonferroni post-hoc (IM/PM vs. RM); *3: *p* < 0.05 after ANOVA and Bonferroni post-hoc (IM/PM compared to NM); *4: *p* < 0.05 after ANOVA and Bonferroni post-hoc (*1/*1 vs *1B/*1B); *5: *p* < 0.05 after ANOVA and Bonferroni post-hoc (*1/*1 vs *1/*1B and *1B/*1B); *6: *p* < 0.05 after ANOVA and Bonferroni post-hoc (TT vs CT and CC); *7: *p* < 0.05 after ANOVA and Bonferroni post-hoc (TT vs CC); *8: *p* < 0.05 after ANOVA and Bonferroni post-hoc (TT/AA vs GG and GT/GA); Underlined: *p* < 0.05 after multivariate analysis (linear regression, which included the following variables: sex, race, CYP2B6 phenotype, CYP2D6 phenotype, CYP1A2*1B, ABCB1 C3435T (rs1045642), G2677 T/A (rs2032582), and C1236T (rs1128503)).

The bold values simply serve to highlight significant results.

*ABCB1* C1236T/T carriers showed lower AUC/DW (unstandardized beta coefficient = −0.072, *p* = 0.047, model *R* ^2^ = 0.151), and Cmax (ANOVA *p* = 0.006 and 0.027, respectively) compared to C/T and C/C carriers; C/C carriers showed lower Cl/F compared to C/T + T/T (unstandardized beta coefficient = −0.072, *p* = 0.045, model *R* ^2^ = 0.155). Moreover, *ABCB1* C3435T T/T carriers showed lower Cmax/DW compared to C/C (ANOVA *p* = 0.021). This association was not confirmed by multivariate analysis. Finally, G2677T/A TT/AA carriers were related to lower Cmax/DW compared to G/G and GT/GA (ANOVA *p* = 0.008 and 0.009, respectively, unstandardized beta coefficient = −0.164, *p* < 0.001, R^2^ = 0.188) (Table 3). The remaining genes (i.e., *CYP2A6*, *CYP2C9*, *CYP2C8*, *CYP2C19*, *CYP3A4*, *CYP3A5*, *UGT1A1*, *SLC22A1*, *SLCO1B1*, and *ABCC2* had no effect on dexketoprofen pharmacokinetic variability, as shown in Table 4.

**TABLE 4:** Description of dexketoprofen pharmacokinetic parameters based on genotypes, haplotypes and phenotypes without statistical significance.

		N	AUC/DW (kg*h*ng/mL*mg)	SD	Cmax/DW (kg*ng/mL*mg)	SD	Tmax (h)	SD	T1/2 (h)	SD	Vd/F (ml/kg)	SD	Cl/F (mL/h*kg)	SD
Other transporters														
*ABCC2* rs717620	CC	46	10,080.33	1,679.14	7,168.66	1,595.02	0.71	0.52	2.23	0.37	325.53	55.55	102.23	17.27
	CT	27	11,146.42	2,507.81	7,391.65	1,609.18	0.70	0.35	2.26	0.33	300.68	45.21	94.18	19.64
	TT	3	10,118.26	622.20	7,593.69	1,386.86	0.64	0.25	2.65	0.77	374.89	91.17	99.22	6.24
*ABCC2* rs2273697	CC	47	10,374.02	1857.48	7,037.95	1,453.91	0.70	0.31	2.25	0.39	318.03	56.10	99.50	16.59
	CT	30	10,676.65	2,214.99	7,545.37	1722.43	0.70	0.62	2.25	0.34	313.40	52.01	98.08	20.75
	TT	7	11,695.04	2,628.54	7,480.65	1797.64	0.55	0.17	2.42	0.13	310.85	63.43	88.92	17.12
*SLC O 1B1* phenotype	NF	53	10,723.96	2,278.01	7,371.42	1,445.13	0.66	0.41	2.29	0.35	317.73	60.30	97.29	18.45
	DF	24	10,416.20	1871.33	7,098.07	1,632.80	0.72	0.51	2.21	0.37	311.58	49.52	99.61	19.57
SLC22A1*2 rs72552763	*1/*1	57	10,610.71	1995.99	7,132.70	1,345.97	0.68	0.44	2.30	0.36	321.07	61.18	97.85	18.36
	*1/*2	18	10,853.95	2,924.00	7,217.74	2,116.74	0.70	0.33	2.17	0.35	298.84	45.34	98.17	23.10
	*2/*2	8	10,290.64	898.75	8,093.09	1,256.08	0.52	0.15	2.17	0.36	303.38	35.95	98.17	8.74
SLC22A1*3 rs12208357	*1/*1	75	10,699.64	2,227.95	7,262.44	1,568.05	0.68	0.42	2.27	0.37	315.42	57.65	97.65	19.41
	*3 carriers	7	10,142.81	822.43	6,875.26	1,344.54	0.57	0.14	2.16	0.27	308.89	49.24	99.31	8.35
SLC22A1*5 rs34059508	*1/*1	81	10,597.41	2,112.73	7,170.16	1,571.49	0.69	0.45	2.27	0.36	316.28	56.27	98.17	18.41
	*1/*5	3	11,699.61	2,780.68	7,987.62	2,164.55	0.61	0.12	2.15	0.22	274.71	42.26	89.68	24.57
Other metabolizing enzymes														
CYP1A2*1C rs2069514	*1/*1	69	10,793.66	2,192.44	7,156.46	1,596.57	0.72	0.47	2.28	0.37	312.79	58.02	96.60	19.10
	*1/*1C	14	10,201.16	1741.31	7,579.92	1721.93	0.54	0.21	2.21	0.28	320.77	51.05	100.96	15.48
	*1C/*1C	2	8,838.25	491.98	7,162.17	307.86	0.62	0.17	1.97	0.09	320.86	3.57	113.48	6.48
CYP1A2*1F rs762551	*1/*1	46	10,696.03	2,125.55	6,947.86	1,608.53	0.75	0.52	2.23	0.33	308.87	53.07	97.41	18.46
	*1/*1F	31	10,562.79	2,220.46	7,562.14	1,503.40	0.62	0.34	2.27	0.37	317.01	54.33	98.68	19.62
	*1F/*1F	8	10,723.98	1917.30	7,526.41	1774.75	0.62	0.17	2.43	0.47	334.93	78.73	95.72	15.77
CYP2A6*9 rs28399433	*1/*1	71	10,707.62	2,153.42	7,215.32	1,596.95	0.72	0.47	2.25	0.36	311.63	56.02	97.24	18.21
	*9 carriers	14	10,358.18	1986.45	7,282.25	1,652.52	0.56	0.20	2.30	0.34	327.83	56.08	100.15	20.33
*CYP3A5* phenotype	EM/IM	14	10,442.31	2,526.85	7,416.15	1866.73	0.91	0.81	2.18	0.34	310.22	52.63	100.50	20.43
	PM	71	10,691.03	2048.01	7,188.91	1,549.96	0.65	0.31	2.28	0.36	315.10	56.99	97.17	18.18
*CYP2C19* phenotype	RM	25	10,695.34	2068.45	6,823.11	1,582.57	0.77	0.50	2.30	0.40	317.78	58.28	97.14	19.29
	NM	36	10,568.11	2,389.82	7,546.70	1,603.81	0.60	0.19	2.26	0.32	318.45	58.76	99.23	20.15
	IM/PM	24	10,725.84	1797.63	7,165.84	1,561.58	0.74	0.59	2.22	0.38	304.43	50.24	96.04	15.30
*CYP2C9* phenotype	NM	54	10,879.35	2,267.47	7,304.83	1716.26	0.73	0.53	2.28	0.36	310.55	54.95	95.83	18.02
	IM/PM	31	10,250.67	1798.62	7,089.62	1,378.94	0.62	0.19	2.23	0.35	320.82	58.19	101.00	19.10
*CYP2C8* haplotype	WT/WT	57	10,896.04	2,267.16	7,282.42	1,552.85	0.72	0.51	2.30	0.38	312.71	55.03	95.87	19.29
	WT/MUT	23	10,300.50	1846.09	6,983.18	1774.51	0.67	0.23	2.18	0.30	314.20	57.81	100.37	17.80
	MUT/MUT	5	9,454.03	525.17	7,705.66	1,329.54	0.48	0.11	2.17	0.38	332.84	68.34	106.53	5.89
CYP3A4*22 *rs35599367*	*1/*1	78	10,650.26	2,102.76	7,320.26	1,544.89	0.66	0.40	2.26	0.33	314.10	54.71	97.62	18.22
	*1/*22	5	10,940.94	2,876.18	7,017.90	1,500.61	0.68	0.25	2.34	0.72	315.97	90.14	97.34	26.51
*UGT1A1 rs887829*	WT/WT	42	10,882.85	2,282.85	7,407.97	1,642.40	0.74	0.49	2.29	0.35	312.85	61.62	95.97	18.71
	WT/MUT	32	10,690.72	1942.70	7,245.28	1,354.80	0.59	0.21	2.21	0.33	304.20	47.38	96.74	16.88
	MUT/MUT	10	9,509.10	1862.20	6,773.76	1806.64	0.65	0.38	2.30	0.51	350.53	48.85	108.84	21.22

NF: normal function; DF: decreased function.

### Safety

All the AEs reported during the three clinical trials were unrelated to dexketoprofen administration. No ADR was reported during any of the three clinical trials. No clinically relevant alteration of vital signs or ECG was observed.

## Discussion

NSAIDs are responsible for 30% of hospitalizations caused by ADRs (Davis and Robson, 2016). These include bleeding, heart attack, stroke or renal damage (Davis and Robson, 2016). The majority of these ADRs can be predicted as they typically occur in vulnerable groups or as a consequence of drug interactions. On March 2020, the Clinical Pharmacogenetics Implementation Consortium published their guideline Nonsteroidal Anti-inflammatory Drugs and *CYP2C9* (Theken et al., 2020). This included, among other, dosing recommendations for ibuprofen based on *CYP2C9*. Thanks to these recommendations, several NSAIDs can be prescribed more safely. Apart from the ethical constraint, avoiding hospitalization saves costs (Davis and Robson, 2016), which contributes to a more sustainable and equitable healthcare system.

Despite belonging to the same NSAID family (propionic acid derivatives) than flurbiprofen and ibuprofen, there is no conclusive evidence that dexketoprofen undergoes CYP (in particular *CYP2C9*) metabolism. Should it undergo this route, it would be secondary, as its primary route of metabolization is glucuronidation. Nor is there much information available on whether this drug is a substrate of solute carrier transporters (SLC) or of the ATP Binding Cassette (ABC) family. Hence, we aimed to explore whether sex, race, *CYP2C9* and other CYP phenotypes as well as polymorphism in relevant pharmacogenes had an impact on dexketoprofen tolerability or pharmacokinetics.

In this work, a mean AUC_∞_ of 4,032 ng*h/mL was reported, which is consistent with previous works (e.g., a mean AUC_∞_ of 4,773 ng*h/ml was reported in a Mexican population (González-Canudas et al., 2019)). Interestingly, AUC_∞_ and Cmax were higher in women than men, however, after DW correction, women showed lower AUC/DW than men and similar C_max_. The first results are expected since women received dexketoprofen to a higher dose-weight ratio as the dose was the same but the weight was lower. To the best of our knowledge, this is the first study that reports a similar association. The sex differences that impact drug pharmacokinetics are well described (Soldin and Mattison, 2009). The differences in transporter or intestinal enzyme gene expression, gastric pH, total body water, blood volume, protein plasma binding patterns, body fat composition, UDP glucuronosyl transferase expression differences or renal elimination rates, among others, could explain this difference in AUC/DW. Nevertheless, the AUC/DW differed in <12% between sexes, therefore the clinical impact may not be relevant. Further studies shall confirm this association. Moreover, the pharmacokinetic differences observed between Latin-Americans and Caucasian group are very small (around 12%) and may be caused by the small sample size or different sex distribution, and, congruently, these differences did not remain significant in multivariate analysis.

One of the greatest challenges when conceiving this work was the strategy for the selection of polymorphisms. On the one hand, the available dexketoprofen pharmacokinetic information was scarce, i.e., it is conjugated predominantly to glucuronide derivatives that are excreted in urine in around 80%. However, little or no information is published on which enzymes perform the glucuronidation and whether dexketoprofen is a substrate of CYP enzymes and of ABC or SLC transporters. On the other hand, no other candidate-gene pharmacogenetic study was published previously and the Pharmacogenomics knowledgebase (PharmGKB) (available at: https://www.pharmgkb.org/chemical/PA166049175) indexes cero clinical or variant annotations for dexketoprofen. Thence, we decided to genotype our healthy volunteers for a set of pharmacogenes that includes important transporters and metabolizing enzymes.

To our knowledge, there is no evidence that dexketoprofen undergoes CYP1A2, CYP2B6, or CYP2D6-mediated metabolism. However, we observed that CYP1A2*1B allele, *CYP2B6* IM/PM and *CYP2D6* IM/PM phenotypes were related to drug accumulation compared to the CYP1A2*1 allele, *CYP2B6* NM/RM and *CYP2D6* NM/UM phenotypes. However, the differences in the exposure (understood as AUC/DW and Cmax/DW) were in all cases <15% and disappeared after multivariate analysis. Therefore, even if these differences are real, they may unlikely have a clinically relevant effect.

Similarly, no study previously demonstrated that dexketoprofen is a substrate of the P-glycoprotein (P-gp, ABCB1), nor its polymorphism was examined in relation to the pharmacokinetic variability of dexketoprofen. Here, the three most relevant *ABCB1* polymorphisms were studied: C1236T, G2677T/A and C3435T (Saiz-Rodríguez et al., 2018). Interestingly, the three mutant alleles, T, A/T and T, respectively, were related to lower Cmax/DW and the association for G2677T/A was confirmed by multivariate analysis, which also showed a relationship between C3435TT allele and lower AUC/DW. Hence, it could be concluded that the presence of mutant alleles in *ABCB1* is related to lower exposure and/or Cmax. This is congruent with previous works, where C3435TT allele was related to decreased risk for nevirapine hepatotoxicity (Ciccacci et al., 2010). In contrast, C3435TT allele was related to decreased fentanyl opioid dose requirements (Horvat et al., 2017) and to increased methotrexate plasma concentrations (Zgheib et al., 2014) compared to the C allele. There is no clear consensus on the clinical effect of *ABCB1* polymorphisms, therefore, further studies are demanded to clarify their impact on dexketoprofen pharmacokinetics. Nevertheless, this is the first study that suggest that dexketoprofen is substrate of the P-gp.

The remaining studied transporter genes (*ABCC2*, *SLC22A1*, and *SLCO1B1*) and metabolizing enzyme genes (*CYP2A6*, *CYP2C19*, *CYP2C9*, *CYP2C8*, *CYP3A4*, *CYP3A5*, and *UGT1A1*) were unrelated to dexketoprofen pharmacokinetic variability, which is consistent with previous knowledge on dexketoprofen pharmacokinetics (Agencia Española del Medicamento y Productos Sanitarios (AEMPS), 2021). The fact that no ADR was reported is consistent with the clinical trial study design (i.e., a single-dose administration of dexketoprofen 25 mg), but it has not allowed us to evaluate the influence of polymorphisms on the safety of this drug.

### Limitations and Strengths

First and foremost, the clinical trial study design did not allow to collect data on: 1) dexketoprofen effectiveness, 2) long-term pharmacokinetics and tolerability. It is possible that UGT2B7 nor UGT2B15 genes are involve in the elimination of dexketoprofen but they could not be evaluated because they were not included in the array used at our center. We were also unable to analyze the influence of Black and Asian race on dexketoprofen pharmacokinetics because there was only one subject of each race. Furthermore, it is important that these results are interpreted with caution given the small sample size. In contrast, bioequivalence clinical trials offer a controlled setting for the evaluation of pharmacokinetic variability based on genetic polymorphism or demographics as confounding factors are avoided. These results can be considered preliminary and further studies are needed to confirm the hypotheses that were raised in the study.

## Conclusion

This candidate gene study is, to our knowledge, the only one published to date (*n* = 85) with a comprehensive genotype screening strategy based on a robust dataset, which lays the foundation for exploring dexketoprofen pharmacogenetics in the future. We conclude that dexketoprofen pharmacokinetics can be influenced by several polymorphisms, although the effect is small and there is not a clear pharmacogenetic predictor that would justify individualization of therapy based on its genotyping. Further studies should be conducted to confirm the role of SNPs in *CYP2B6*, *CYP2D6*, *CYP1A2*, and *ABCB1* on the pharmacokinetic variability of dexketoprofen. Current evidence on dexketoprofen pharmacogenetics does not justify its inclusion in pharmacogenetic guidelines.

## Data Availability

The raw data supporting the conclusions of this article will be made available by the authors, without undue reservation. The data is the property of the promoter and will be made available upon reasonable request.

## References

[B1] Agencia Española del Medicamento y Productos Sanitarios (AEMPS) (2021). Enantyum - Dexketoprofen Drug Label.

[B2] Aguirre C. García M. (2016). Causality Assessment in Reports on Adverse Drug Reactions. Algorithm of Spanish P System. Medicina Clínica (English Edition) 147, 461–464. 10.1016/j.medcli.2016.06.012 27450163

[B3] Barbanoj M.-J. Antonijoan R.-M. a. Gich I. (2001). Clinical Pharmacokinetics of Dexketoprofen. Clin. Pharmacokinet. 40, 245–262. 10.2165/00003088-200140040-00002 11368291

[B4] Caudle K. E. Rettie A. E. Whirl-Carrillo M. Smith L. H. Mintzer S. Lee M. T. M. (2014). Clinical Pharmacogenetics Implementation Consortium Guidelines for CYP2C9 and HLA-B Genotypes and Phenytoin Dosing. Clin. Pharmacol. Ther. 96, 542–548. 10.1038/clpt.2014.159 25099164 PMC4206662

[B5] Caudle K. E. Sangkuhl K. Whirl‐Carrillo M. Swen J. J. Haidar C. E. Klein T. E. (2019). Standardizing *CYP 2D6 G*enotype to Phenotype Translation: Consensus Recommendations from the Clinical Pharmacogenetics Implementation Consortium and Dutch Pharmacogenetics Working Group. Clin. Transl Sci. 13, 116. 10.1111/cts.12692 31647186 PMC6951851

[B6] Ciccacci C. Borgiani P. Ceffa S. Sirianni E. Marazzi M. C. Altan A. M. D. (2010). Nevirapine-Induced Hepatotoxicity and Pharmacogenetics: A Retrospective Study in a Population from Mozambique. Pharmacogenomics 11, 23–31. 10.2217/pgs.09.142 20017669

[B7] Davis A. Robson J. (2016). The Dangers of NSAIDs: Look Both Ways. Br. J. Gen. Pract. 66, 172–173. 10.3399/bjgp16X684433 27033477 PMC4809680

[B8] Desta Z. Gammal R. S. Gong L. Whirl‐Carrillo M. Gaur A. H. Sukasem C. (2019). Clinical Pharmacogenetics Implementation Consortium (CPIC) Guideline for CYP2B6 and Efavirenz‐Containing Antiretroviral Therapy. Clin. Pharmacol. Ther. 106, 726–733. 10.1002/cpt.1477 31006110 PMC6739160

[B9] González-Canudas J. García-Aguirre L. J. Medina-Nolasco A. Ruíz-Olmedo M. I. Medina Reyes L. J. Zambrano Tapia L. (2019). Bioequivalence Evaluation of Two Oral Formulations of Dexketoprofen-Trometamol (Solution and Tablets) in Healthy Subjects: Results from a Randomized, Single-Blind, Crossover Study. Trends Med. 19. 10.15761/TiM.1000176 PMC6667897 29749718

[B10] Horvat C. M. Au A. K. Conley Y. P. Kochanek P. M. Li L. Poloyac S. M. (2017). ABCB1 Genotype Is Associated with Fentanyl Requirements in Critically Ill Children. Pediatr. Res. 82, 29–35. 10.1038/pr.2017.103 28388599 PMC5509475

[B11] Karch F. E. Lasagna L. (1977). Toward the Operational Identification of Adverse Drug Reactions. Clin. Pharmacol. Ther. 21, 247–254. 10.1002/cpt1977213247 837643

[B12] Ramsey L. B. Johnson S. G. Caudle K. E. Haidar C. E. Voora D. Wilke R. A. (2014). The Clinical Pharmacogenetics Implementation Consortium Guideline for SLCO1B1 and Simvastatin-Induced Myopathy: 2014 Update. Clin. Pharmacol. Ther. 96, 423–428. 10.1038/clpt.2014.125 24918167 PMC4169720

[B13] Saiz-Rodríguez M. Belmonte C. Román M. Ochoa D. Jiang-Zheng C. Koller D. (2018). Effect of ABCB1 C3435T Polymorphism on Pharmacokinetics of Antipsychotics and Antidepressants. Basic Clin. Pharmacol. Toxicol. 123, 474. 10.1111/bcpt.13031 29723928

[B14] Saiz-Rodríguez M. Ochoa D. Belmonte C. Román M. Vieira de Lara D. Zubiaur P. (2019). Polymorphisms in CYP1A2, CYP2C9 and ABCB1 Affect Agomelatine Pharmacokinetics. J. Psychopharmacol. 33, 522–531. 10.1177/0269881119827959 30789308

[B15] Scott S. A. Sangkuhl K. Stein C. M. Hulot J.-S. Mega J. L. Roden D. M. (2013). Clinical Pharmacogenetics Implementation Consortium Guidelines for CYP2C19 Genotype and Clopidogrel Therapy: 2013 Update. Clin. Pharmacol. Ther. 94, 317–323. 10.1038/clpt.2013.105 23698643 PMC3748366

[B16] Soldin O. P. Mattison D. R. (2009). Sex Differences in Pharmacokinetics and Pharmacodynamics. Clin. Pharmacokinet. 48, 143–157. 10.2165/00003088-200948030-00001 19385708 PMC3644551

[B17] Theken K. N. Lee C. R. Gong L. Caudle K. E. Formea C. M. Gaedigk A. (2020). Clinical Pharmacogenetics Implementation Consortium Guideline (CPIC) for CYP2C9 and Nonsteroidal Anti‐Inflammatory Drugs. Clin. Pharmacol. Ther. 108, 191–200. 10.1002/cpt.1830 32189324 PMC8080882

[B18] Zgheib N. K. Akra-Ismail M. Aridi C. Mahfouz R. Abboud M. R. Solh H. (2014). Genetic Polymorphisms in Candidate Genes Predict Increased Toxicity with Methotrexate Therapy in Lebanese Children with Acute Lymphoblastic Leukemia. Pharmacogenet. Genomics 24, 387. 10.1097/FPC.0000000000000069 25007187

[B19] Zubiaur P. Saiz-Rodríguez M. Ochoa D. Belmonte C. Román M. Mejía G. (2019). Influence of CYP2B6 Activity Score on the Pharmacokinetics and Safety of Single Dose Efavirenz in Healthy Volunteers. Pharmacogenomics J. 20, 235. 10.1038/s41397-019-0103-3 31628422

[B20] Zubiaur P. Saiz-Rodríguez M. Ochoa D. Navares-Gómez M. Mejía G. Román M. (2020). Effect of Sex, Use of Pantoprazole and Polymorphisms in SLC22A1, ABCB1, CES1, CYP3A5 and CYP2D6 on the Pharmacokinetics and Safety of Dabigatran. Adv. Ther. 37 (8), 3537–3550. 10.1007/s12325-020-01414-x 32564268

